# Thyroid Hormone and Estrogen Regulate Exercise-Induced Growth Hormone Release

**DOI:** 10.1371/journal.pone.0122556

**Published:** 2015-04-13

**Authors:** Daniele Leão Ignacio, Diego H. da S. Silvestre, João Paulo Albuquerque Cavalcanti-de-Albuquerque, Ruy Andrade Louzada, Denise P. Carvalho, João Pedro Werneck-de-Castro

**Affiliations:** 1 Institute of Biophysics Carlos Chagas Filho and School of Physical Education and Sports, Federal University of Rio de Janeiro, Rio de Janeiro, Brazil; 2 Institute of Biophysics Carlos Chagas Filho, Federal University of Rio de Janeiro, Rio de Janeiro, Brazil; 3 Department of Endocrinology and Metabolism, Rush University Medical Center, Chicago, Illinois, United States of America; Universidad Pablo de Olavide, Centro Andaluz de Biología del Desarrollo-CSIC, SPAIN

## Abstract

Growth hormone (GH) regulates whole body metabolism, and physical exercise is the most potent stimulus to induce its secretion in humans. The mechanisms underlying GH secretion after exercise remain to be defined. The aim of this study was to elucidate the role of estrogen and pituitary type 1 deiodinase (D1) activation on exercise-induced GH secretion. Ten days after bilateral ovariectomy, animals were submitted to 20 min of treadmill exercise at 75% of maximum aerobic capacity and tissues were harvested immediately or 30 min after exercise. Non-exercised animals were used as controls. A significant increase in D1 activity occurred immediately after exercise (~60%) in sham-operated animals and GH was higher (~6-fold) 30 min after exercise. Estrogen deficient rats exhibited basal levels of GH and D1 activity comparable to those found in control rats. However, after exercise both D1 activity and serum GH levels were blunted compared to sedentary rats. To understand the potential cause-effect of D1 activation in exercise-induced GH release, we pharmacologically blocked D1 activity by propylthiouracil (PTU) injection into intact rats and submitted them to the acute exercise session. D1 inhibition blocked exercise-induced GH secretion, although basal levels were unaltered. In conclusion, estrogen deficiency impairs the induction of thyroid hormone activating enzyme D1 in the pituitary, and GH release by acute exercise. Also, acute D1 activation is essential for exercise-induced GH response.

## Introduction

Overweight and obesity are epidemic diseases leading to diabetes and metabolic syndromes, and are associated with cardiovascular disorders [1], especially for women facing the menopause transition [2]. Fluctuations in sex hormones at different stages of reproductive life, such as menarche, pregnancy, and menopause transition, may play a role in the adipose tissue expansion. Notably, menopausal transition is associated with unfavorable changes in body composition, abdominal fat deposition and general health outcomes [3–5]. Thus, understanding the mechanism involved in the genesis of obesity during the menopausal transition will help the development of strategies to fight obesity [4].

Growth hormone (GH)/insulin-like growth factor-1 (IGF-1) axis regulates growth and development during childhood and adolescence, but also regulates body composition, metabolism and exercise aerobic capacity throughout life [5]. Increased lipolysis and free fat acids (FFA) mobilization are the main effect of GH in metabolism [6]; e.g. the most prominent effect of physiological GH pulse exposure is a marked stimulation of lipolysis [7–9]. Its deficiency (GHD) is associated with increased body fat and a lower lean body mass [10, 11]. These changes in body composition are associated with metabolic derangements including insulin resistance [9].

Besides GH, estrogen (E2) and thyroid hormones (TH) control energy expenditure and are essential for body weight balance [12]. Active thyroid hormone T3 acts virtually in all body tissues trough interaction with nuclear receptors. In addition to serum T3, local availability and clearance are well controlled by types I, II, and III iodothyronine deiodinases (D1, D2, and D3, respectively) via removal of specific iodine atoms from the precursor molecule T4 or T3 itself. D1 and D2 catalyze the 5'-deiodination of T4 and therefore they are considered activating enzymes due to subsequent T3 production. On the other hand, D3 inactivates T4 and T3 through their 5-deiodination [13,14]. Thus, modulation of deiodinases expression and activity customize T3 biological effects. Moreover there is an interplay by E2 and TH in several biological effects, for example, the lack of E2 in rats leads to obesity and decreases D2 activity in brown adipose fat in a long-term ovariectomy protocol (9 weeks) [11].

Multiple physiological factors modulate GH secretion, such as age, estrogen, nutrition, sleep, body composition, regional distribution of body fat, stress, insulin, fitness, TH and physical exercise [12]. Among them acute aerobic exercise is the most potent stimulus to GH release [15–19]. Indeed, after exercise GH levels increase 5.1-fold in humans [18] and GH secretion is correlated to exercise intensity [15,19]. Proposed mechanisms for such increase are: acidosis [20]; central adrenergic outflow [21], core body temperature [22] and cholinergic mediation [23]. Interestingly, obese patients present a blunted spontaneous GH secretion as well as in response to exercise attributed to a decrease in pulsatile GH release and a shorter half-life of endogenous GH [24]. Many obesity-related physical adaptations resemble those recognized in GH-deficient adults [16] and menopause [4], including reduced muscle mass and exercise capacity, increased body fat especially abdominal visceral fat (AVF), and increased cardiometabolic risk [16] suggesting the involvement of these hormones in the genesis of obesity.

GH is a major T3-induced protein in the rat pituitary [25] and T3 regulates GH gene in transcriptional and posttranscriptional level [26, 27]. Somatotrophs highly express D1 and are the major cell population of pituitary [25, 28]. Pituitary (PIT) D1 [28] and brown adipose tissue (BAT) D2 [11] activities increase after a single running exercise session [28], and in response to a 8-week swimming exercise training program [11]. In the later study, both PIT D1 and BAT D2 activities response to exercise are blunted in trained obese estrogen deficient rats [11]. Taken together, these results suggest that impairment of T4-to-T3 conversion in PIT and BAT by decreased D1 and D2 activities respectively in response to exercise could be involved in the genesis of obesity in E2 deficient rats trough (i) decreasing GH release by PIT and/or (ii) reducing BAT metabolism. Here we report that low levels of E2 prejudice PIT D1 activation and GH release after acute high intensity treadmill exercise before the onset of obesity (short term ovariectomy). In addition, pharmacological blockage of D1 also decreases exercise-induced GH release linking thyroid hormone signaling to GH release after exercise.

## Materials and Methods

### Animals and experimental groups

Adult female Wistar rats weighing 200–250 g donated by the Vital Brazil Institute (Niteroi, 113 RJ—Brazil) were kept in a temperature-controlled (22±1°C) animal room, with a 12 h light-dark cycle (lights on at 6 pm), and pelleted commercial chow (Paulínea, São Paulo, Brazil; iodine content 2 mg/kg) and water were available *ad libitum*. All adult female rats showed a regular 4–5 day estrous cycle monitored by vaginal cytology collected each morning for 2 consecutive weeks before starting the experiments. The Rio de Janeiro Federal University Institutional Committee for Animal Use in Research (CEUA—CCS—EEFD 05) approved this study, which was in accordance with the International Guiding Principles for Biomedical Research Involving Animals (Geneva, Switzerland).

### Ovariectomy and exercise protocol

The surgery consisted of bilateral removal of ovaries under anesthesia with ketamine (50 mg/kg, i. p.) and xylazine (5 mg/kg, i. p.) as describe previously [11] and all efforts were made to minimize suffering. Sham (Sh) animals were submitted to the surgical procedure except to ovaries removal. The animals were used after 10 days of ovariectomy in order to prevent changes in body composition that occurs later on.

All animals were submitted to an exercise protocol consisted of 20 min treadmill running at 70–75% of maximum speed S_max_ under a constant slope of 10°. To determine S_max_, the animals were adapted to run 2–3 days at 17 cm/s during 5 min. After adaptation period, each rat was submitted to a maximal exercise test, as previously described [28]. Trial began at a starting velocity of 17 cm/s and a constant slope of 10°. Treadmill velocity was then increased by 2 cm/s every 2 min and the rats ran until exhaustion (when the animals stayed in the steel grids despite increasing shocks stimuli). S_max_ was considered if animals completed at least 75% of the stage. Mean room temperature was maintained at 22°C and a stainless steel grid at the end of the treadmill provided an electrical stimulus to keep the rats running. All tests and exercise protocol were carried out with a motor-driven treadmill chamber (Panlab—LSI Letica model). S_max_ correspond to speed at maximal oxygen consumption [29].

Animals were not fed after the exercise session and were killed by decapitation and blood was collected for hormone concentration analyses. Serum was obtained after centrifugation of the blood at 1500 x g for 20 min and stored at -80°C. Pituitary were dissected out and stored at -80°C until processing for enzymatic measurements. Retroperitoneal and inguinal fat pads were completely removed, weighted for evaluation of adiposity and discarded.

### Body composition

Body composition was evaluated by dual-energy X-ray absorptiometry. Before submitted to DXA, animals were anesthetized with ketamine (50 mg/kg, i. p.) and xylazine (5 mg/kg, i. p.) as describe previously [11]. Lunar DXA 200368 GE instrument (Lunar, Wisconsin, USA) with specific software (encore 2008, version 12.20 GE Healthcare) was used to measure: Body weight (g), body fat mass (g), lean body mass (g), bone mineral density (g/cm2), fat free mass (g), visceral fat (%) and total body fat (%).

### Serum hormone measurements

Serum estradiol levels were measured by specific RIA (MP Biomedicals, LLC, USA). The intra-assay variation coefficient was 3.5–15.7%. The inter-assay variation coefficient was 5.5–15.4%. Radioactivity was counted in an automatic gamma counter (1470 Wallac WizardTM automatic gammacounter). Limit of detection was 10–3 000 pg/ml. The results were expressed in pg/ml. All the procedures were carried out following the recommendations of the kits.

Serum total GH (ng/ml) was measured by either specific radioimmunoassay (RIA—Lincon Research) or ELISA (Mediagnost, Tuebingen, Germany), as stated in figure legends. All immune assays were performed according to manufacturer’s instructions. Limit of detection was 0.5–50 ng/ml for RIA and 0.025–1.5 ng/ml for ELISA. The intra- and inter-assay coefficient of variation was 3.5–15.7% and 5.5–15.4%, respectively (limits of detection 10–3000 pg/ml. Radioactivity was counted in an automatic gamma counter (1470 Wallac WizardTM automatic gamma counter).

### Type 1 iodothyronine deiodinase (D1) activity

Pituitary gland was homogenized in 0.1 M sodium phosphate buffer containing 1 mM EDTA, 0.25 M sucrose, and 10 mM dithiothreitol (pH 6.9). Protein concentration was measured by the Bradford method [30]. Homogenates (20 μg of protein) were incubated for 1 h at 37°C with 1 μM rT3 (Sigma, USA) containing freshly purified (Sephadex LH20) tracer (100.000 cpm [^125^I] rT3, PerkinElmer Life Sciences, Boston, MA, USA) and 10 mM dithiothreitol in 100 mM PBS containing 1 mM EDTA (pH 6.9), as previously described [31]. Blank incubations were carried out in the absence of protein. The reaction was stopped on ice followed by immediate addition of 200 μl fetal bovine serum (Cultilab, BR) and 100 μl trichloroacetic acid (50%, v/v). The specific enzymatic activity was expressed as picomoles of rT3 deiodinated/min/mg protein.

### PTU treatment

Propylthiouracil (PTU) acutely inhibits D1 activity [31]. 100 μl of PTU (*ip;* 2 mg of PTU/100g bw) was injected 4 h and 5 min before the acute exercise session into intact animals. The control group received 100 μl of saline.

### Statistics

The results are expressed as mean ± SEM and p < 0.05 was considered statistically significant. GH secretion in intact animals was analyzed by one-way ANOVA followed by Dunnett’s Multiple Comparison test. GH secretion in ovariectomized rats and D1 activity were analyzed by two-way ANOVA followed by Bonferroni multiple comparison test. Serum levels of estradiol, GH after D1 inhibition body and fat mass were analyzed by unpaired t-test.

## Results

### Short-term ovariectomy (Ovx) decreases E2 levels but does not induce obesity

Ovx is a well-recognized model to study estrogen deficiency in rodents [11, 32, 33] and E2 deficient rats gain weigh after 9–14 d of Ovx [33] and develop obesity [11,32]. We studied rats submitted to a short-term Ovx (10 d) to avoid the impact of obesity on GH secretion [16,24]. Indeed, Ovx rats exhibited lower levels of estradiol (33%) compared to intact rats, but the body weight was not different (Table 1). Moreover, short-term Ovx did not modify body composition (Table 1). In order to rule out the effects of Ovx on exercise capacity we tested the groups before and after Ovx. Ovx rats ran as much as control animals and reached comparable levels of maximal speed and blood lactate levels (Table 2). All animals were able to complete the acute exercise session. The speed (75% S_max_) employed in acute exercise was similar between groups as well as the blood lactate increment (Table 3).

**Table 1:** Effects of 10 days of ovariectomy on hormone concentration and body composition.

**Parameter**	**Sham (n = 9–20)**	**Ovx (n = 9–20)**
Estradiol (pg/ml)	59.55±4.59	39.94±3.73 **
Body weight (g)	219.7±3.25	228.3±4.09
Inguinal fat (g)	0.85±0.04	0.71±0.07
Retroperitoneal fat (g)	0.43±0.03	0.44±0.02
Total body fat (%)	30.08±2.95	29.42±2.94
Visceral fat (%)	28.57±2.80	28.08±3.02
Lean body mass (g)	145.44±14.34	146.69±11.92
Fat free mass (g)	152.33±14.31	162.92±12.24
Fat mass (g)	62.33±5.55	65.38±6.81
Bone mineral density (g/cm^2^)	0.14±0.01	0.13±0.01

Ovx—rats submitted to ovariectomy for 10 days.

** p<0.01 compared to sham group

**Table 2:** Effects of 10d of ovariectomy (Ovx) on maximal aerobic capacity and blood lactate concentration.

	**Time (min)**		**Smax (m/min)**		**[La] (mmol/L) after Ovx**	
	**Before**	**After Ovx**	**Before**	**After Ovx**	**Basal**	**After Max Test**
**Sham**	33.2 ± 2.15	32.0 ± 1.94	30.1 ± 1.2	28.7 ± 1.2	2.9 ± 0.5	8.1 ± 0.3 *
**Ovx**	29.0 ± 1.57	30.8 ± 1.6	27.9 ± 1.1	29.1 ± 0.9	1.8 ± 0.1	7.0 ± 0.4 *

S_max_—maximal speed (n = 18); [La]—lactate concentration (n = 5)

* p<0.05 compared to Basal group

**Table 3:** Characteristic of the acute exercise session.

	**Speed (75% of S**_max_ **)**	**[La] (mmol/L) after Ovx**	
		**Basal**	**After**
**Sham (n = 6)**	21.6 ± 0.9	1.8 ± 0.5	6.6 ± 1.17 *
**Ovx (n = 6)**	21.9 ± 0.6	2.0 ± 0.3	6.6 ± 1.72 *

Ovx—rats submitted to ovariectomy for 10 days; S_max_—maximal speed; [La]—lactate concentration

* p<0.05 compared to Basal group

### High intensity exercise induces GH secretion

**Fig 1:**
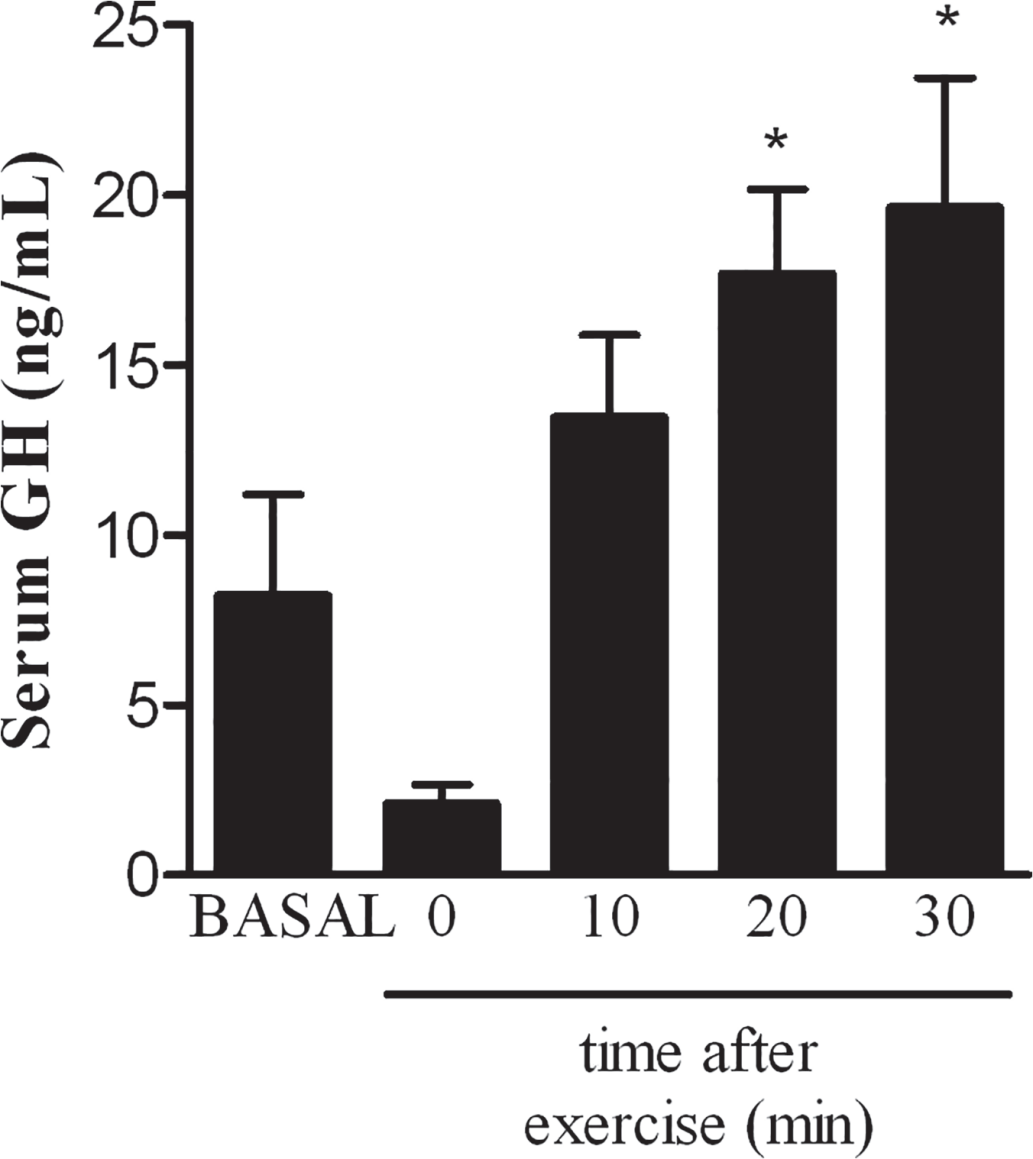
Post-exercise growth hormone secretion. Values are mean ± SEM (n = 10). *p<0.05 vs basal (non-exercised group) analyzed by one-way analysis of variance followed by the Dunnett’s multiple comparison test.

In humans, after acute high intensity exercise growth hormone increases in blood and continues to increase during the following recovery minutes [34]. In rats, only one study investigated GH response to acute exercise [35]. Thus, we first performed a time course of GH release in our model of high intensity treadmill exercise. Immediately after exercise, serum GH levels decreased (~74%) although not statistically significant (Fig 1). Twenty minutes after exercise, serum GH increased 2-fold and peaked at 30 min (~2.4-fold) (Fig 1). For that reason, subsequent studies were performed at 30 minutes.

### Estrogen deficiency impairs acutely exercise-induced pituitary D1 activation and GH release

**Fig 2:**
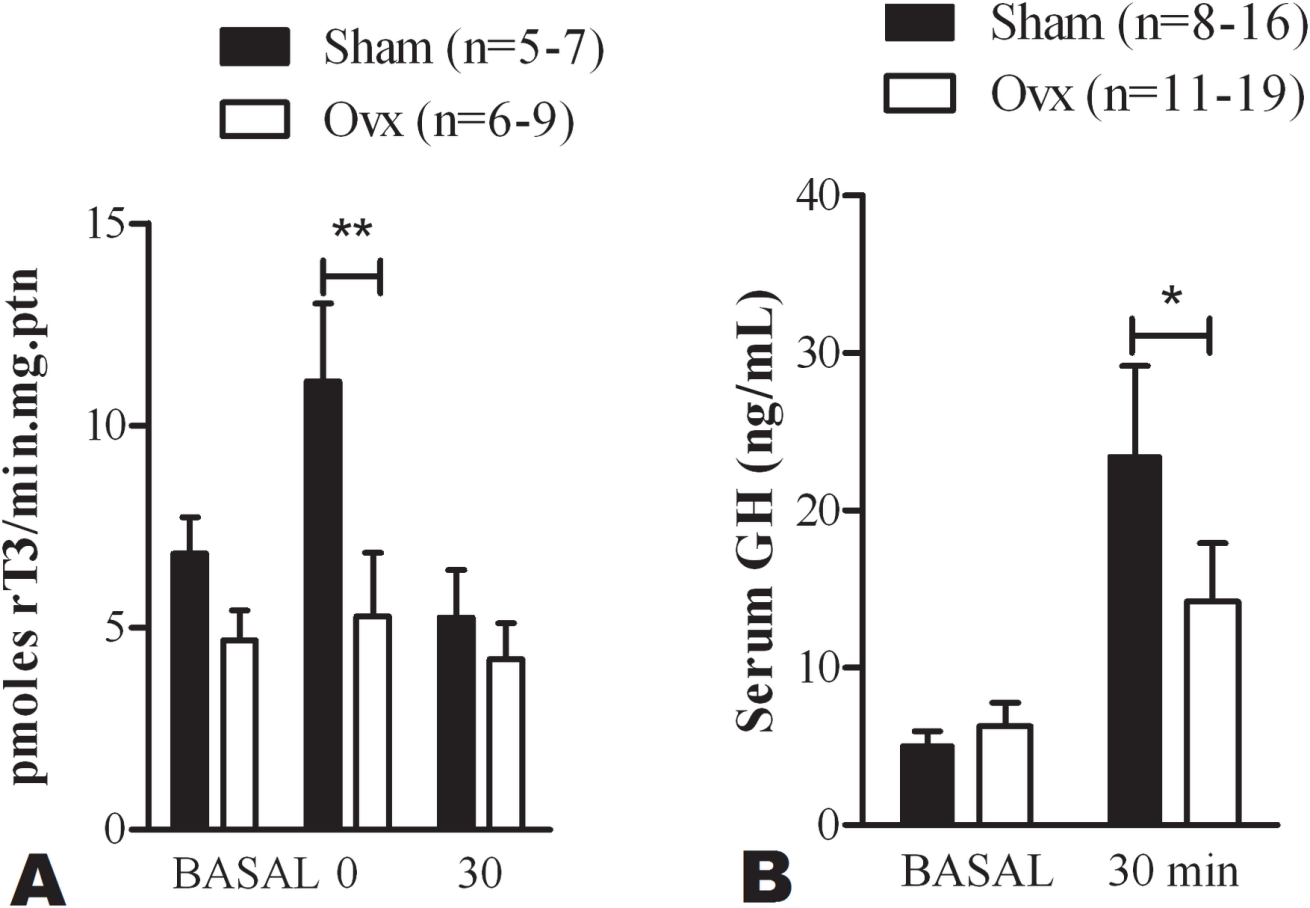
Effects of high intensity treadmill exercise on pituitary D1 activity and GH release. **A)** Pituitary type 1 deiodinase activity in Sham and Ovx groups before (basal) and after exercise (30 min) and **B)** Serum levels of growth hormone before (basal) and after exercise (30 min). Values are mean ± SEM. Sample size is shown in parenthesis. *p<0.05 and ** p<0.01 analyzed by two-way ANOVA followed by the Bonferroni multiple comparison test.

Both D1 and D2 activate thyroid hormones by converting T4-to-T3 locally [13]. PIT D1 activity is greater in exercised rats but not D2 [28], strongly suggesting a role played by D1 in PIT function after exercise. Thus, we tested whether Ovx could impair D1 activation in PIT after acute exercise, as reported by our group after 8 weeks of swimming exercise training [11]. A significant increase in D1 activity occurred immediately after exercise (~60%; Fig 2A) in sham-operated animals and GH was higher (~6-fold; Fig 2B) 30 min after exercise. Estrogen deficient rats exhibited comparable basal levels of GH and D1 activity. However, after exercise both D1 activity and serum GH levels were blunted in Ovx compared to sedentary rats (Fig 2A and 2B).

### Inhibition of D1 activity decreases exercise-induced GH release

**Fig 3:**
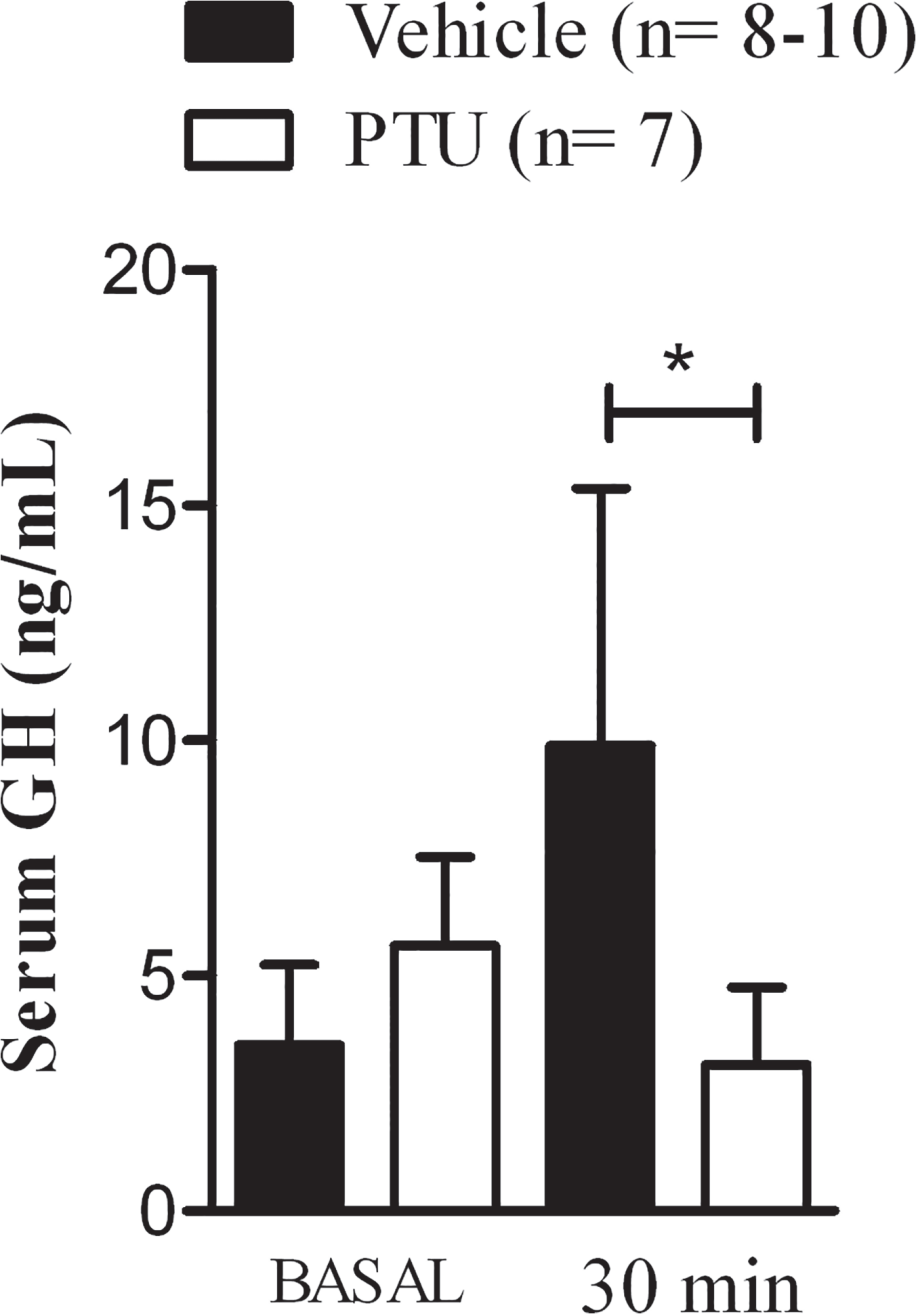
Impact of D1 inhibition by PTU on circulating levels of growth hormones. Values are mean ± SEM. Sample size is shown in parenthesis. *p<0.05 analyzed by two-way ANOVA followed by the Bonferroni multiple comparison test.

The fact that E2 impairs D1 and GH response to exercise suggest that D1 could be important to GH release after exercise. Thus, it is conceivable to hypothesize that inhibition of D1 could blunt GH response to exercise. To test this, intact rats were treated with PTU, a pharmacological blocker of D1 activity [31] before submitted to the exercise protocol. Remarkably, PTU abolished GH appearance in blood 30 min after exercise (Fig 3), suggesting that pituitary D1 activation is important for local T3 production and GH release after exercise.

## Discussion

The present study reveals that estrogen deficiency caused by short-term ovariectomy (Ovx) blunts growth hormone (GH) release induced by high intensity exercise (Fig 2B), as well as thyroid hormone activating enzyme D1 activity in pituitary (Fig 2A). In this model, female rats are not obese yet (Table 1) and exercise capacity is preserved (Table 2) which allows us to study the early events of Ovx before obesity is installed, since overweight impacts GH response to exercise [23]. These set of experiments suggest that T4-to-T3 conversion in PIT could play a role in GH release after exercise. Indeed, pharmacological blockage of D1 abolished GH induction in our model of high intensity treadmill exercise in female rats (Fig 3).

Few studies investigated GH secretion using physical exercise stimulus in rats [12,35]. Therefore, we first determined the time course of GH secretion after high intensity treadmill exercise in female rats (Fig 1). Although not significant, exercise seems to suppress GH release right after exercise (time 0 in Fig 1; q = 1.648) to ~26% of basal levels. Thereafter, as in humans [15], GH increased with time reaching a peak at 30 minutes after exercise (Fig 1). Butkus *et al* [35] studying female Sprague-Dawley rats reported that GH release decreased during the acute treadmill exercise bout and typical GH pulses did not recover up to 5.5 hours post-exercise. Their exercise protocol did not increase lactate levels [35]. The rat strain used and the exercise intensity could explain the contrasting results. In our study, we set the intensity (75%) relative to maximum aerobic capacity (maximal speed—S_max_) to reproduce studies performed in humans [15] and this intensity increased lactate levels in blood more than 3-fold (77–88% of maximal lactate response in average) confirming the high intensity nature of our exercise protocol. Also, we previously showed that 75% S_max_ in male Wistar rats represented 88% of maximal oxygen consumption which characterizes a high intensity exercise. In humans, the linear dose-response relationship between exercise intensity and the GH response is well stablished [19]. Felsing *et al* [15] studying the effects of the exercise intensity on GH circulating levels in men reported greater serum GH and insulin concentrations 20–30 min after the highest exercise intensity. Therefore, our exercise protocol is a potent stimulus to induce GH appearance in blood, as occurs in humans.

Many factors modulate GH secretion such as physical exercise, fat mass and gonadal steroids [12]. Basal levels of GH are diminished in obesity as well as GH response to exercise [36]. Reduction in the quantity of GH secreted per pulse is the primary mechanism responsible for the lower serum GH concentrations after exercise in obese compared to lean women [24]. The lack of ovarian hormones in female adult rats leads to a significant increase in body mass [11] between 9–13 days after Ovx and the effects are reverted by Estrogen (E2) treatment [32,33]. To avoid the influence of obesity-induced impairment of GH secretion we did the short-term ovariectomy protocol (10 d), when Ovx rats were not obese yet (Table 1) although serum E2 levels were 33% reduced (Table 1). In our model, GH secretion after 30 minutes of exercise was lower in ovariectomized rats compared to sham, suggesting that estradiol is important to exercise-induced GH release (Fig 2B). It is already known that E2 regulates GH axis [36–38]. Our results add new insight in GH modulation under a stressful condition as intense physical exercise.

Thyroid hormone (TH) remarkably modulates GH secretion [12,14,25]. Tissue concentration of T3, the biologically active TH, and saturation of its nuclear receptors rely on transmembrane transport from blood to cytoplasm and the locally conversion of T4 to T3 by deiodinases [14]. Both D1 and D2 activate TH in pituitary, and 50% of PIT is comprised by GH-secreting cells. We previously demonstrated that both acute and chronic exercise positively modulate D1 activity in PIT, potentially linking T4-to-T3 conversion to GH release. [11,28]. Swimming training during 8 weeks increased D1 activity in sham-trained female rats, while gonadectomy blunted the exercise training effect [11]. A time-course study in male rats using the same acute exercise model reported that PIT D1 activity increased 30, 60, and 120 min after exercise compared to sedentary control animals [28]. In the present study we confirmed that D1 activity is induced by acute exercise at 30 min in female rats, however, after 60 min the activity returned to basal levels (Fig 2A). The reason for this sexual dimorphism is unclear. More importantly, the lack of E2 blunts D1 response after exercise suggesting that E2 is critical to T3 production through D1 activation in PIT. Together, the evidence that Ovx impairs both GH release and PIT D1 activity induction led us to investigate the role of D1 in exercise-induced GH release. Notably, inhibition of D1 by PTU injection abolished GH response (Fig 3).

In conclusion, estrogen deficiency impairs the induction of thyroid hormone activating enzyme D1 in the pituitary and GH release by acute exercise. Also, pituitary D1 activation is essential for exercise-induced GH response.
